# Supplementary figures and images for: Therapeutic effects of adenosine in high flow 21% oxygen aereosol in patients with Covid19-pneumonia
Source: PLoS One. 2020 Oct 8;15(10):e0239692. doi: 10.1371/journal.pone.0239692 (PMC7544127; doi:10.1371/journal.pone.0239692)

**
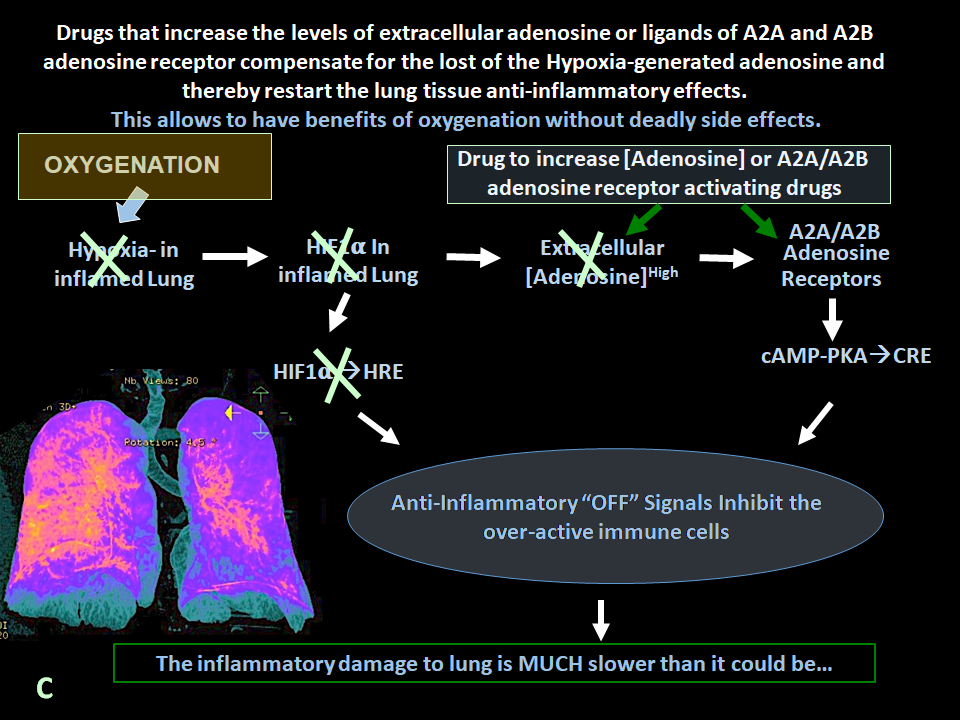

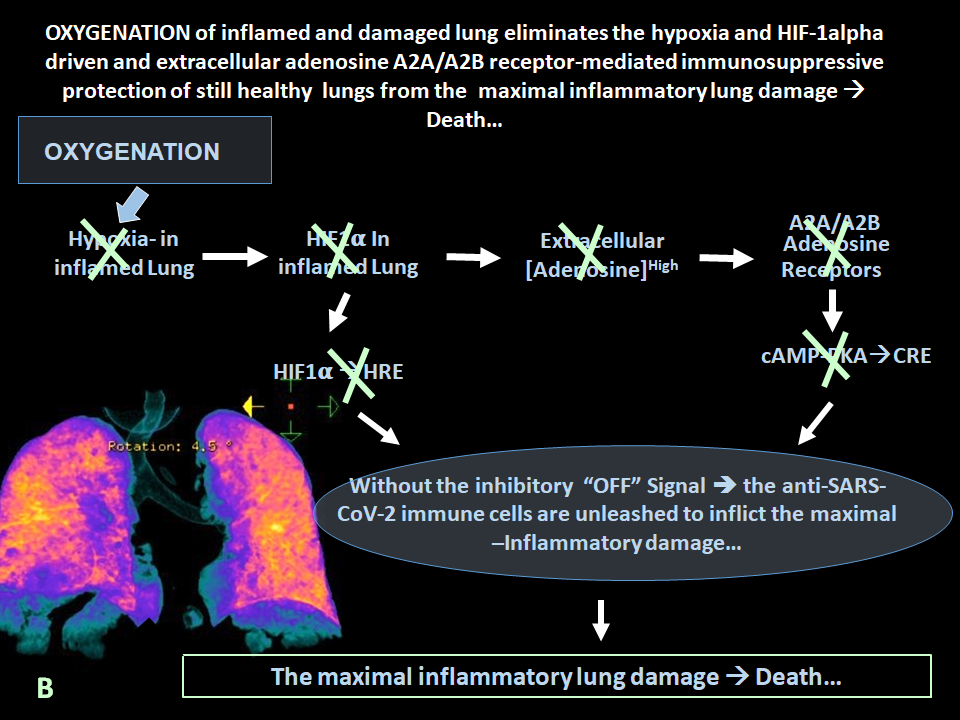

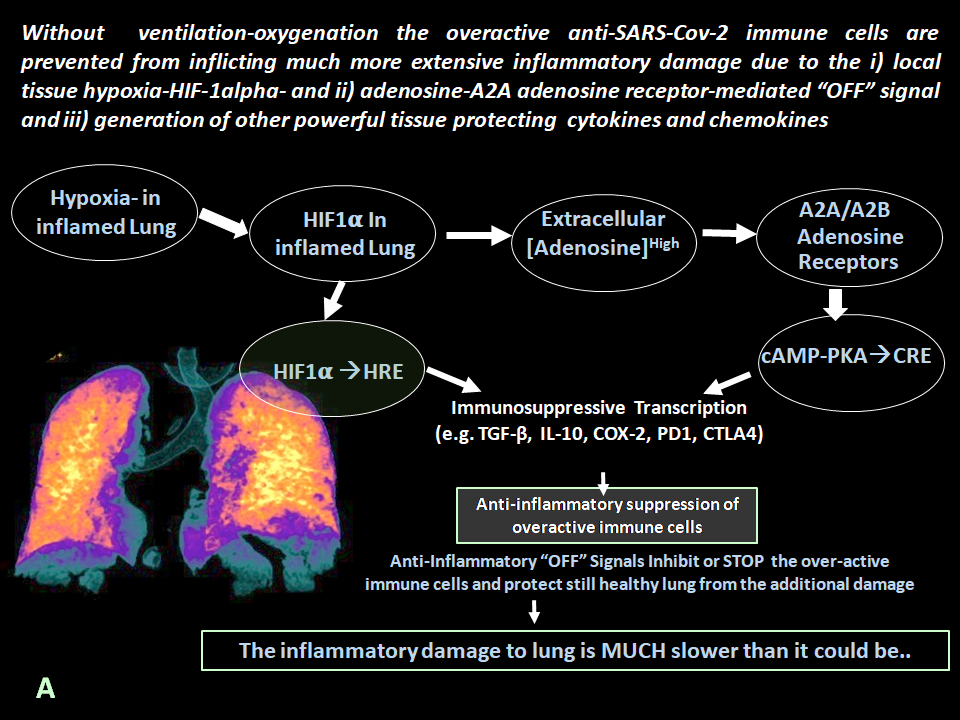
S1 Fig**

Supplement: S1 Fig — (DOCX) [file pone.0239692.s001.docx]
